# Are committee experiences of minoritized family medicine faculty part of the minority tax? a qualitative study

**DOI:** 10.1186/s12909-023-04848-3

**Published:** 2023-11-13

**Authors:** Kendall M Campbell, Stacy A. Ogbeide, Angela Echiverri, Gina Guillaume, Johnathan E Henderson, Nicole Jackson, Crystal M Marquez, Carolina Miranda, Melissa Montoya, Keyona Oni, Grant Pierre, Afi Mansa Semenya, LaTraia Scott, Victoria Udezi, Valerie J Flattes, José E Rodríguez, Judy C Washington

**Affiliations:** 1 University of Texas Medical Branch, Texas, USA; 2 University of Texas Health Sciences Center at San Antonio, San Antonio, USA; 3 Contra Costa Health Services, Contra Costa County, USA; 4 North by Northeast Community Health Center, Portland, USA; 5 Floyd Family Medicine Residency Program, Rome, USA; 6 Boston Medical Center, Boston University, Boston, USA; 7 SUNY Downstate Medical Center, Brooklyn, USA; 8 Montefiore Bronx Health Collective, Bronx, USA; 9 Texas Tech Paul L. Foster School of Medicine, El Paso, USA; 10 Carolinas Healthcare System, Charlotte, USA; 11 University of Massachusetts Medical School, Boston, USA; 12 Uniformed Services University, Bethesda, USA; 13 University of Texas Southwestern Medical School, Dallas, USA; 14 University of Utah Health, 26 S 2000 E, 5750B EHSEB, 84112 Salt Lake City, UT USA; 15 Atlantic Health System, New Jersey, USA

**Keywords:** African americans, American indians, Hispanic or latino, Health Occupation, Faculty

## Abstract

**Background and Objectives:**

Because much of the work in academic medicine is done by committee, early career URiM faculty, are often asked to serve on multiple committees, including diversity work that may not be recognized as important. They may also be asked to serve on committees to satisfy a diversity “check box,” and may be asked more often than their non-URiM peers to serve in this capacity. We sought to describe the committee experiences of early career URiM faculty, hypothesizing that they may see committee service as a minority tax.

**Methods:**

Participants in the Leadership through Scholarship Fellowship (LTSF) were asked to share their experiences with committee service in their careers after participating in a faculty development discussion. Their responses were analyzed and reported using qualitative, open, axial, and abductive reasoning methods.

**Results:**

Four themes, with eight sub-themes (*in parenthesis*), emerged from the content analysis of the LTSF fellows responses to the prompt: Time commitment (*Timing of committee work and lack of protected time for research and scholarship*), URiM Committee service (*Expectation that URiM person will serve on committees and consequences for not serving*), Mentoring issues (*no mentoring regarding committee service, faculty involvement is lacking and the conflicting nature of committee work*) and Voice (*Lack of voice or acknowledgement*).

**Conclusions:**

Early career URiM faculty reported an expectation of serving on committees and consequences for not serving related to their identity, but other areas of committee service they shared were not connected to their URiM identity. Because most of the experiences were not connected to the LTSF fellows’ URiM identity, this group has identified areas of committee service that may affect all early career faculty. More research is necessary to determine how committee service affects URiM and non-URiM faculty in academic family medicine.

## Introduction

The minority tax, a term used to describe extra duties assigned to faculty members from minority groups underrepresented in medicine (URiM), is a phenomenon that is often seen in medical schools and has been described in the literature [1–4]. Because institutions have an interest in having URiM input, constant requests for committee membership to URiM faculty can be burdensome for the individuals involved and leave them less time for academic and clinical work [2]. Thus, increased levels of committee service may be an element of the minority tax that plays a role in the retention and advancement of URiM faculty [1].

Due to talent loss, attrition, and issues of faculty equity, the resulting low numbers of underrepresented academic medical faculty have been discussed extensively in the medical literature [5–7]. Faculty who are underrepresented in medicine (URiM) include people who identify as Black or African American, Latinx (Hispanic or Latino), American Indian and Alaska Native (AIAN), Native Hawaiian and other Pacific Islander, and Southeast Asians. The minority tax, the gate blocking of underrepresented minority faculty, high junior faculty attrition, and the persistent net of factors that hinder URiM and women faculty advancement are major causes for these low numbers [1, 4, 8, 9].

While there is evidence describing the effect of the minority tax on URiM faculty, [2, 10, 11], there has not been any literature addressing how committee service impacts the careers of these faculty. This manuscript describes committee service as experienced by early career URiM faculty, using qualitative methods to analyze reflections composed after discussing that topic. We hypothesized that early career URiM academic family physicians would share experiences that point to committee service being seen as a “minority tax.“ [1–4, 12] This manuscript is a qualitative analysis of URiM early career academic family physicians’ responses to a question about committee service. This research has the potential to inform institutional leaders on how committee service is seen and informs URiM faculty about the possible effects of committee service on their individual career success and advancement.

## Methods

This study was deemed exempt by the University of Utah institutional review board, IRB # 0091384, as part of a departmental educational umbrella IRB exemption. Twelve early career URiM academic family medicine fellows participating in the Society of Teachers of Family Medicine (STFM) Leadership through Scholarship Fellowship (LTSF) were asked to reflect on their experiences with committee work in their academic faculty careers. LTSF is a URiM-focused fellowship designed to help URiM faculty navigate the minority tax [13, 14]. It is a supportive fellowship which has been described elsewhere in the literature [13, 14]. LTSF fellows were selected after a nationwide application process involving a committee consisting of the LTSF faculty and STFM administrators. As LTSF is advertised as URiM faculty development, all twelve participants in the group were assistant professors or instructors, identified as members of a race/ethnic minoritized group [(75% Black or African American, 16.7% Latinx (Hispanic or Latino), and 8.3% Southeast Asian)].

During a faculty development session for the LTSF fellowship, faculty gathered with a former medical school dean for mentorship and advice. The former dean shared their perspective on promoting URiM faculty success and encouraged fellows to share their personal stories of being URiM faculty and growth and advancement in academic medicine. Comments from the LTSF fellows included experiences in their home institutions, experiences with mentorship, and interactions with institutional leadership. Significant time was spent speaking about committee service and its role in an academic medical career.

The dean who served as a mentor identifies as a white man. As is typical with URiM faculty, each of the LTSF fellows were among a handful of URiM faculty at their institutions, and many of them were the only ones in their department. The fellows were appointed to serve on departmental and college/school wide committees, but the fellows understood that service did not fulfill promotion requirements. Fellows expressed that they were the only URiM faculty in each of the committees on which they served, and because they are all early career faculty, most of them were new to the institution where they were serving.

After a robust discussion of many experiences that the medical school dean and the fellows shared in academic medicine, fellows were (re)introduced to the concept of “minority tax” and how it affects URiM faculty and institutions. This meeting was held virtually, and all the LTSF fellows (A.E., G.G., J.H., N.J., C.M., C.M., M.M., K.O., G.P., A.M., L.S., V.U.) and two of the faculty (J.C.W. and K.M.C.) participated in the discussion.

After the meeting, the faculty met and decided that there was more to be learned from the fellows regarding committee service. Fellows were then verbally asked to free text a paragraph outlining their committee experiences since beginning their careers in academic medicine. Reflection on this and other fellowship activities are integral parts of the fellowship curriculum. Fellows were intermittently reminded via email or group text to complete their paragraphs. LTSF fellows knew this was part of a qualitative research project and were interested in sharing their experiences. The LTSF fellows and faculty decided that all involved would be authors of the paper and that the fellows’ collective views would be valuable addition to the literature.

The responses to the above prompt were analyzed through rigorous qualitative methods, starting with grounded theory. Grounded theory lends itself to the creation of theory after data is analyzed. Grounded theory analysis allows the researcher to explore the data inductively, to develop codes through a series of steps, and then to finalize the codes for analysis [15–17]. Once the steps and analysis are complete theories may be generated regarding URiM and the committee tax. The qualitative analyst did not have contact with participants during data collection. The analysis was conducted after the training ended and after all reflections were received. The text was then analyzed and then coded. A textual analysis of the data could assist the conference coordinators in reviewing common threads and ease the review of central themes from the dataset.

The text was first read several times by the qualitative researcher (VF) and then open-coded inductively, allowing themes to emerge from the data using techniques described by Corbin, Strauss, and Saldaña [15–17]. Finally, a process of selective coding was conducted to look at the relationships between the coding and categories/themes. Through a process called member checking, fellows were able to review the results for accuracy and truthfulness. The fellows, however, were unaware if everyone was participating, nor did they see what their peers wrote until after the qualitative analysis of their experiences. LTSF fellows (A.E., G.G., J.H., N.J., C.M., C.M., M.M., K.O., G.P., A.M., L.S., V.U.) each contributed their experiences with committees, and they participated in the writing of the introduction and the discussion. V.F. conducted the qualitative analysis, and J.C.W., K.M.C., and J.E.R. participated in the delivery of the content in the LTSF and the production of the manuscript. LTSF fellows also reviewed this manuscript before submission.

## Results

All LTSF fellows (Table 1) shared their experiences with committee service in writing, an average response of 234 words. All fellows agreed with the table and the themes. We hypothesized that early career URiM academic family physicians would share experiences that point to committee service being seen as a “minority tax,” and found that the themes identified by the qualitative researchers confirmed our hypothesis. Table 1 presents the results of the analysis of the responses, with themes, sub-themes, and data exemplars. It also serves as a description of committee service as experienced by the fellows in the study.

**Table 1:** Themes, sub-themes, and data exemplars from participants reflections on committee service

Theme	Sub-theme	Data exemplars
Time Commitment	Timing of Committee Work	“…because of my involvement in these numerous committees, my scholarly work suffered,” “Heavy burden on me as an underrepresented minority in medicine” “Committees take time and dedication and can be burdensome if you are not ready or willing to be an active participant on the committee.” “There is great tension between my desire to engage with the community I serve (and belong to) and my need to attend to my clinical duties in such a way that allows time for scholarship and my personal ambitions.” “Does not receive an adequate allotment of protected time (if any), and (2) is not accounted for in promotion tracks.”
	Lack of protected time for research and scholarship	“Stretched myself too thin.” “Does not receive an adequate allotment of protected time (if any)” “My concern is that most of these commitments do not come with supportive time outside of academic time or count much towards promotion.”
URiM Committee Service	Expectation that the URiM person will serve on committees	“Although no one tells you to get involved, you are either thrust into a committee by default of your position, or it is covertly expected that you get involved if you want your voice heard.”
	Consequences of not serving	“My first recalled experience with committee tax began in residency. This was prior to eventual gained understanding that specific committee involvement was key for advancement, but far along enough to know a refusal [to serve on a committee] would be frowned upon.”
Mentoring Issues	No mentoring regarding committee service	“Looking back, more involvement from the remainder of our faculty members, mentorship for publishing into academic journals, and use of committee involvement as a criteria for promotion and tenure would have helped to life the burden of the ‘committee tax’ from me as a URM.” “In addition, with the demands from these committees, what gets lost is ample time for mentorship and development to ensure clinical, education, and scholarly output is maximized and sustained.”
	Faculty involvement is lacking	“I did not feel equipped with the tools and resources needed to publish my work in academic journals in part, due to a lack of oversight and mentorship by faculty.” “Out of a group of 15 core faculty there was only a consistent group of 3–4 who served on these committees, and they frequently had minimal time to meet in their schedule due to other clinical/academic commitments.”
	The conflicting nature of committee work	“…my currency in academic medicine is my voice and how I use it to effect change and dismantle barriers that keep those that are historically marginalized and systemically excluded from thriving and experiencing joy and being celebrated in these spaces of learning and healing.” “Ultimately, participation in these numerous committees with only a few core faculty at the expense of scholarly work led to fatigue and burnout as the completion of one committee task was met with the onset of another committee’s takeoff.” “I later learned as a faculty member, when it came to promotion and advancement, scholarly activity carried more weight.” “…it does not contribute to my ability to move further along in my career trajectory or up the proverbial academic ladder.”
Voice	Lack of Voice or Acknowledgement	“This work defines me, and it also breaks me because this labor goes by unnoticed, unappreciated, and unacknowledged.” “Even though I was invited to these committees and told that my unique and diverse input was highly valued, I had no role in final decision making. I was there for a figure piece, and I was there to make everyone in the room feel more comfortable.” “We are often asked to join a committee, but our voice is not always heard, listened to or taken seriously.” “I am still at Instructor level. I am pouring so much of myself and my talents into improving our department and institution, and this work is not recognized by promotions”

Four themes, with eight sub-themes (*in parenthesis*), emerged from the content analysis of the LTSF fellows responses to the prompt: Time commitment (*Timing of committee work and lack of protected time for research and scholarship*), URiM Committee service (*Expectation that URiM person will serve on committees and consequences for not serving*), Mentoring issues (*no mentoring regarding committee service, faculty involvement is lacking and the conflicting nature of committee work*) and Voice (*Lack of voice or acknowledgement*). Data exemplars are included in Table 1. Fellows also expressed that committee work involved skills and attitudes that are not intuitive and should be taught.

## Discussion

Three of the four broad themes (Time commitment, Mentoring issues, and Voice) are likely common among all new faculty in academic medicine [18, 19]. However, the theme of URiM Committee Service is unique to the identities of the LTSF fellows. In addition, six of the eight sub-themes (*Timing of Committee Work, Lack of protected time for research and scholarship, No mentoring regarding committee service, Faculty involvement is lacking, The conflicting nature of committee work, and Lack of Voice or Acknowledgement*) illustrate some of the difficult experiences and thoughts early career faculty face in academic family medicine. These challenges are not limited to family medicine, are present in academic medicine,[1] and in faculty careers that do not have clinical responsibilities [20]. The sub-themes *Expectation that the URiM person will serve on committees* and the *Consequences of not serving* represent issues faced solely by URiM faculty. These identified sub-themes are consistent with the literature that characterize the experiences of URiM faculty in academic medicine and are consistent with the minority tax [1, 4, 11, 21]. These “identity” sub-themes also indicate that the LTSF fellows are conscious of their URiM identities and how that can affect how their work is perceived. Because most of the themes and sub-themes are not unique to URiM faculty, these findings signal that more research is needed on how committee work fits into an academic career.

Most of the fellows identify as URiM and women, and their comments, although not specifically stated reflect their individual intersectionality. The inability to be heard, and their over representation in committee assignments, are consistent with the minority woman tax and the citizenship tax described elsewhere in the literature that specifically affects women [4, 21]. In addition, URiM women experience this combination of taxes is not additive, but multiplicative, increasing the burdens on this important group of faculty [4].

There are a few limitations to this work. First, this is a group of highly motivated academic family physicians participating in a faculty development activity designed to increase their scholarly output and share successful leadership strategies, introducing a strong bias towards the prioritization of research and scholarship in their careers. This introduces some bias in that committee service was not their principal reason for participating in the LTSF fellowship. LSTF fellows have been prolific in scholarship after participating in the program, with 31 publications now associated with this effort. Next, a Hawthorne effect is likely present in this analysis,[22] as the fellows knew their responses would be analyzed and published. While it is difficult to predict how a Hawthorne effect may manifest, it is reasonable to expect that, in a group of new URiM faculty being taught by senior URiM faculty who were the first authors to characterize identity taxation in academia as “minority tax” in academic medicine, [1, 2, 4, 11] the LTSF fellows would have spoken more about the uneven distribution of committee service work and attributed the reason for this uneven distribution to their identities. In most of the themes and sub-themes, no association was made between the LTSF fellows’ identities and their experiences working on committees. It is very difficult, if not impossible, to eliminate the Hawthorne effect in qualitative research [23]. Conversely, it is surprising that in the environment where this research was conducted, more connection between identity and committee experiences was not made. Finally, the written nature of the responses may have limited the number of words the fellows shared.

It is important to discuss the topic of researcher representation in this project, also known as the “involvement paradox.” [24] Having LTSF fellows participate in the research as subjects encourages field proximity, given that this is a topic directly impacting the faculty fellows – an important element of qualitative research. To establish the trustworthiness of the data collected, however, a level of professional distance can also be beneficial to decrease any contamination of the research outcomes. The involvement paradox and field proximity bring value to this article by representing the authors’ experiences as URiM faculty honestly. The involvement paradox allows researchers to acknowledge that this experience exists as well as gives the reader an opportunity to appreciate the lens through which the knowledge from this paper was composed.

This work is in concordance with the existing literature, which shows that faculty members serve on between 0 and 8 committees and that URiM faculty in academic medicine may have more service requirements than their peers [10, 18, 25]. This paper adds to the literature by describing the committee service activities of *URiM family medicine* faculty and their thoughts about this service. Recent articles on academic promotion do not address the increased committee burden on URiM faculty leading to potential negative effects on their career [26–30]. All authors of this study recognized that committee service is important and want their contributions and opinions to be valued and utilized [31]. Knowing how motivated, career-minded URiM faculty see committee service could be beneficial in making it a more effective tool in the department and institutional governance, increasing its value to individual participants in the committees. These findings represent the experiences of URiM early career faculty in academic family medicine. More research is necessary to determine the impact of committee service on non-URiM early career faculty, regardless of identity or specialty.

## Data Availability

The raw data are available upon request from Valerie J Flattes, PhD. Valerie.flattes@nurs.utah.edu.
